# Femtosecond Laser Assisted Crystallization of Gold Thin Films

**DOI:** 10.3390/nano11051186

**Published:** 2021-04-30

**Authors:** Ayesha Sharif, Nazar Farid, Rajani K. Vijayaraghavan, Patrick J. McNally, Gerard M. O’Connor

**Affiliations:** 1 National Centre for Laser Applications (NCLA), School of Physics, National University of Ireland Galway, H91 TK33 Galway, Ireland; nazar.farid@nuigalway.ie (N.F.); gerard.oconnor@nuigalway.ie (G.M.O.); 2 I-Form, the SFI Research Centre for Advanced Manufacturing, National Centre for Laser Applications (NCLA), School of Physics, National University of Ireland Galway, H91 TK33 Galway, Ireland; 3 I-Form, the SFI Research Centre for Advanced Manufacturing, Advanced Processing Technology Research Centre, School of Electronic Engineering, Dublin City University, Glasnevin, Dublin 9, D09 V209 Dublin, Ireland; rajani.vijayaraghavan@dcu.ie (R.K.V.); patrick.mcnally@dcu.ie (P.J.M.)

**Keywords:** femtosecond laser, laser wavelength, crystallinity, laser fluence, gold thin films, damage threshold, sheet resistance, two temperature model

## Abstract

We propose a novel low temperature annealing method for selective crystallization of gold thin films. Our method is based on a non-melt process using highly overlapped ultrashort laser pulses at a fluence below the damage threshold. Three different wavelengths of a femtosecond laser with the fundamental (1030 nm), second (515 nm) and third (343 nm) harmonic are used to crystallize 18-nm and 39-nm thick room temperature deposited gold thin films on a quartz substrate. Comparison of laser wavelengths confirms that improvements in electrical conductivity up to 40% are achievable for 18-nm gold film when treated with the 515-nm laser, and the 343-nm laser was found to be more effective in crystallizing 39-nm gold films with 29% improvement in the crystallinity. A two-temperature model provides an insight into ultrashort laser interactions with gold thin films and predicts that applied fluence was insufficient to cause melting of gold films. The simulation results suggest that non-equilibrium energy transfer between electrons and lattice leads to a solid-state and melt-free crystallization process. The proposed low fluence femtosecond laser processing method offers a possible solution for a melt-free thin film crystallization for wide industrial applications.

## 1. Introduction

Gold (Au) is a material of great technological significance due to its interesting associated properties, for example high electrical conductivity, chemical inertness, good stability and biocompatibility [1,2]. Crystalline Au thin films and nanoparticles with low defect densities offer potential applications especially in microfluidic devices [3]; electrochemical sensing [4]; biochemical sensing [5]; and as transparent conductors [6]. Such applications require higher electrical conductivity which is sensitive to any change in size and distribution of grains in Au thin films. Annealing is a widely accepted method to enhance the electrical as well as structural properties of thin films through a heat treatment process for desired applications. Conventional annealing methods using a rapid thermal [7]; furnace [8]; flame [9]; and oven annealing [10] have been reported for Au thin films on various substrates to improve the grain size and their structural properties. However, such annealing methods have limitations in terms of long processing durations and high process temperatures which are incompatible for substrates with low melting points. Moreover, the non-selectivity of a particular region for localized annealing is an issue as higher temperatures can damage the other nearby components, often leading to contamination and degradation of electrical performance [11]. Laser annealing is versatile and rapid; it offers precise and localized energy distribution to the material and is reported for Au films using continuous wave [12] and pulsed nanosecond lasers [13,14]. However, long, and short pulse interaction causes material melting, followed by a cooling and a re-solidification process [15]. Therefore, for thin film crystallization, a non-melt and low temperature process is desirable as it enables the structural and material properties to be altered without additional complications caused by melting.

Ultra-short laser pulses (≤picosecond) are of great importance since their pulse durations are much shorter than the duration of major electron–lattice relaxation processes resulting in a non-equilibrium two-temperature phenomenon in the material [16,17]. By using this concept, we demonstrate how a femtosecond (fs) laser enables crystallization in Au thin films at a fluence lower than the damage threshold. Such low fluences are insufficient to cause any melting since the lattice temperature remains below the melting temperature of Au; this was confirmed from the two-temperature model (TTM) simulation findings. Ultra-short laser pulse crystallization has been reported by many research groups, especially for crystallizing semiconductor thin films [18,19,20,21,22]. In this paper, we present low fluence annealing of Au thin films deposited on a quartz substrate by using fs laser pulses of three different wavelengths. Our results are promising and exhibit a decrease in sheet resistance and hence an improved crystallinity of films after laser scanning. To the best of our knowledge, this is the first report on the melt-free crystallization of Au thin films with ultra-short laser pulses at fluences below the damage threshold.

## 2. Materials and Methods

**Figure 1:**
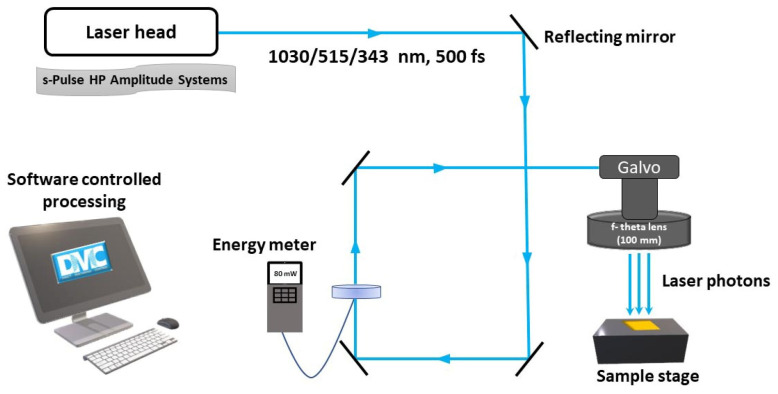
The schematic diagram of the laser setup used for the crystallization experiment.

The Au thin films of thicknesses 18-nm and 39-nm were sputter coated (Emitech K550X, Lambda Photometrics Ltd, Hertfordshire, United Kingdom) on a 500 µm thick quartz substrate and film thicknesses were confirmed with a high-resolution atomic force microscope (Agilent 5000, Agilent Technologies UK Ltd, Berkshire, United Kingdom). The coating current was kept at 25 mA and coating time was varied from 4 min to 8 min to obtain 18-nm and 39-nm film thicknesses, respectively. The standard deviation in sheet resistance of coated samples is within the acceptable limit; ±0.40 Ω/Sq for the 18-nm and ±0.14 Ω/Sq for the 39-nm film, respectively. A fs laser (s-Pulse HP, Amplitude systems, Pessac, France, with IR (1030 nm), green (515 nm) and UV (343 nm) wavelengths is employed for the crystallization of Au thin films. The fs laser with a pulse duration of 500 fs is operated at 100 kHz repetition rate. The laser is focused with a telecentric f-theta lens with 100 mm focal length. The sample position is controlled with a 3D computer-controlled stage (Aerotech 3200, Aerotech, Inc, Pittsburgh, PA, USA). A galvanometer XY scanning system (SCANLAB, hurry SCAN II, SCANLAB GmbH, Puchheim, Germany) controls the pulse to pulse overlap on the sample and the schematic of experimental setup is shown in Figure 1. A four-point probe station (Ossila T2001A2, Ossila Limited, Sheffield, United Kingdom) is used to measure the sheet resistance before and after each laser scan on the sample. To confirm the crystallization, X-Ray diffraction (XRD) was performed using a Jordan Valley Bede D1 high resolution XRD system with a copper (λ = 1.5405 Å) radiation source operated at 45 kV and 40 mA. The transmittance spectra are obtained with a UV-VIS spectrophotometer (SHIMADZU UV-2600, SHIMADZU CORPORATION, Kyoto, Japan) to investigate the effect on optical properties of laser crystallized films. A theoretical study was conducted to interpret the laser-Au thin film interactions by considering the Au-quartz interface into the model. A multiphysics finite element method (FEM) simulation based on TTM is carried out by using COMSOL Multiphysics^®^ software (COMSOL Inc., version 5.5, Burlington, MA, USA).

## 3. Results and Discussion

A single pulse damage threshold fluence $\left(\phi_{th}\right)$ was determined experimentally using Liu’s method [23];

(1) $$D^{2}=2\omega_{o}^{2}ln\left(\frac{\phi_{o}}{\phi_{th}}\right)$$

where *D* is the measured diameter of the crater, *ω_o_* is the Gaussian beam waist radius, $\phi_{o}$ is peak fluence which is calculated by $\phi_{o}=2E_{P}/\pi \omega_{o}^{2}$. $E_{P}$ is the applied laser energy and $\phi_{th}$ is the damage threshold, the value of fluence at which Au film surface starts to damage. The experimentally calculated parameters for 18-nm and 39-nm Au films on quartz substrates are provided in the Table 1.

### 3.1. Damage Threshold Fluence ($\phi_{th}$) Measurements

**Figure 2:**
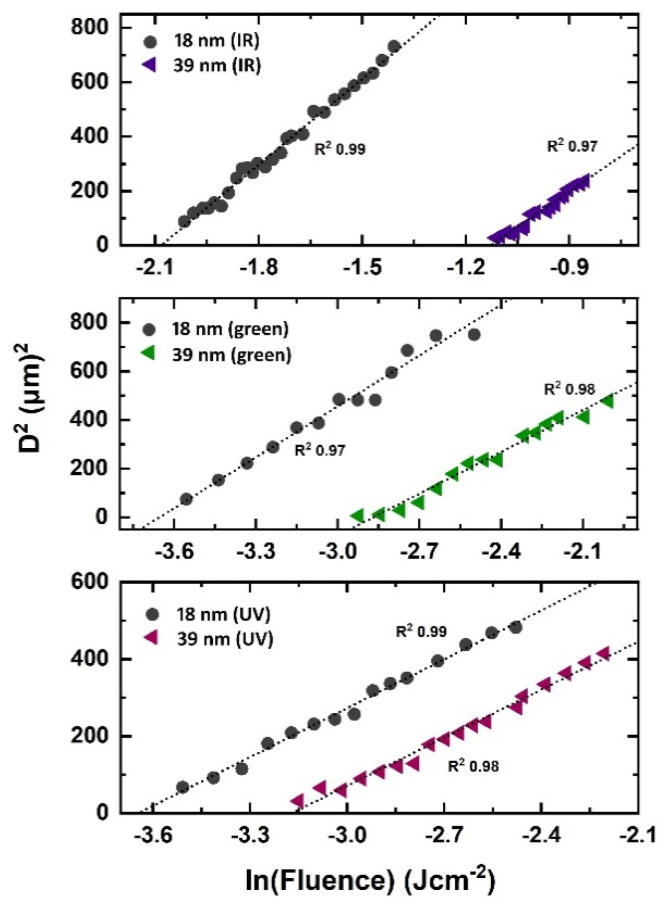
Plots between natural log of applied fluence and squared crater diameter for 18-nm and 39-nm Au films on quartz substrate for IR, green and UV laser wavelengths, respectively.

**Figure 3:**
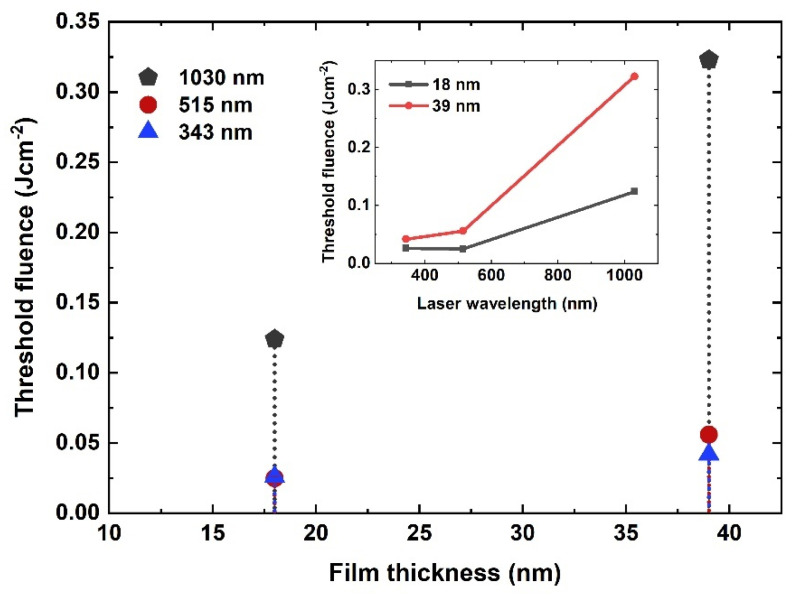
A comparison of threshold fluence with film thickness. The inset shows the variation in threshold fluence with employed laser wavelengths for 18-nm and 39-nm thick Au films, respectively.

Figure 2 represent plots showing the linear relationship between the natural log of the applied fluence and the squared diameter of laser crater for both 18-nm and 39-nm Au films obtained for IR, green and UV laser, respectively. The single pulse damage threshold fluence is calculated by extrapolating the curves in Figure 2. In the femtosecond time domain, energy absorption by electrons and succeeding hot electrons diffusion governs the energy deposition. This diffusion of electrons can be described by characteristic penetration depth (*L_c_*) which is a measure of the diffusion length of hot electrons into the material within the hot electron gas before the electron–phonon relaxation occurs [25]. *L_c_* can exceed the optical penetration length (*l_opt_*) significantly which defines how deep the laser beam can penetrate into the material. *l_opt_* is calculated by using the optical constants from [24] for Au as 14.59 nm, 20.27 nm and 12.50 nm for UV, green and IR laser, respectively. The crucial parameter controlling the depth of hot electrons is electron-phonon coupling parameter; thereby governing energy loss into the material and the zone of thermal damage [26]. For 18-nm Au films, $\phi_{th}$ is calculated as 0.124 ± 8.11 × 10^−4^ Jcm^−2^, 0.025 ± 7.07 × 10^−4^ Jcm^−2^ and 0.026 ± 5.3 × 10^−4^ Jcm^−2^ for IR, green and UV laser wavelengths, respectively. The measured $\phi_{th}$ for 39-nm thick film is as 0.323 ± 8.11 × 10^−4^ Jcm^−2^, 0.056 ± 8.37 × 10^−4^ Jcm^−2^ and 0.042 ± 3.8 × 10^−4^ Jcm^−2^ for IR, green and UV laser wavelength, respectively. The variation in $\phi_{th}$ with film thickness and applied laser wavelengths is shown in Figure 3. It is noted that threshold fluence, to create damage, increases with increasing film thickness. It is consistent for all used wavelengths such as IR, green and UV laser pulses. It is observed that for films thinner than a characteristic optical penetration length, the damage threshold fluence is a linear function of film thickness. It increases linearly with film thickness under (*d_film_* < *L_c_*) and saturates when film becomes thick as compared to electron diffusion length reported in [25,26].

A comparison of estimated $\phi_{th}$ with wavelengths for 18-nm and 39-nm Au films deposited on quartz substrate is given in the inset of Figure 3. Highest threshold for damage is obtained for IR and then for green- and UV laser-treated Au films. For 18-nm film, $\phi_{th}$ for green wavelength is 0.025 Jcm^−2^, which is close to UV laser wavelength (0.026 Jcm^−2^), respectively. Higher *l_opt_* for green results in deeper transmission of photons into the film and thereby increase in the threshold fluence. Moreover, these interesting wavelength dependent variations in $\phi_{th}$ are attributed to response of Au electronic system (5d^10^6s^1^) to photon energies associated with wavelength. In Au, optical absorption typically occurs due to free carriers via interband and inverse bremsstrahlung absorption [27]. The minimum energy (eV) needed to excite 5d band electrons into 6s band is defined as the interband transition threshold (ITT). For Au, ITT varies across the Fermi surface from 1.84 to 2.4 eV [28,29,30]. Photons with energies greater than ITT for example, green (2.4 eV) and UV (3.61 eV) significantly initiate the interband absorption via 5d electrons. This higher absorption of laser energy at UV and green laser wavelengths caused the damage at lower threshold fluence compared to IR. For IR (1.19 eV), the absorption is only by the 6s band electrons via intraband absorption, and the d-band electrons are unperturbed. Therefore, a greater number of photons are required to produce any change, i.e., the damage on the surface of film.

### 3.2. Conductivity Measurements

**Figure 4:**
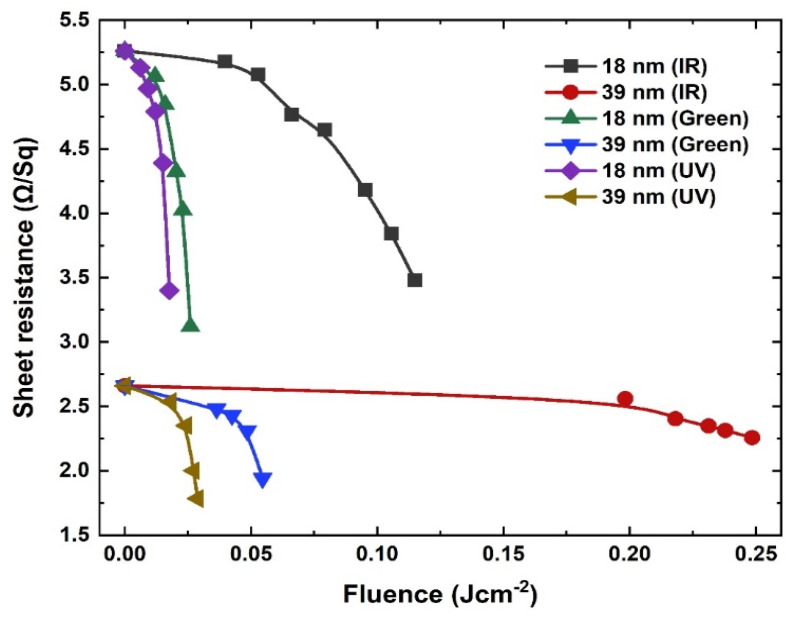
Decrease in sheet resistance as a function of applied laser fluence in an optimized regime before and after IR, green and UV laser scanning of 18-nm and 39-nm Au films, respectively.

Laser crystallization is greatly affected by the processing parameters, i.e., laser wavelength, pulse duration, scan speed and the repetition rate. Au thin films are scanned with high overlapped laser pulses at low scan speeds and at very low fluences to avoid melting and any surface damage. To enable crystallization, Au thin films were scanned at 100 kHz repetition rates and with 5-µm hatching. The repetition rate and scan speed (180 mms^−1^) were kept constant for all three wavelengths which gave a ~96% overlap with 24.39 shots per area (SPA) for IR, ~95% pulse overlap with 19.86 SPA for green and 93.8% overlap for UV with 16.12 SPA, respectively. The process repeatability is confirmed for a number of samples and representative examples are shown in Figure 4. Normalized electrical sheet resistance values are observed to decrease after scanning with highly overlapped pulses in an optimized fluence regime. The sheet resistance dropped from 5.22 ± 2.45 × 10^−3^ to 3.45 ± 2.12 × 10^−3^ Ω/Sq for 18-nm film and from 2.38 ± 2.31 × 10^−3^ to 2.02 ± 1.9 × 10^−3^ Ω/Sq for 39-nm film after scanning with IR laser pulses. Similarly, sheet resistance decreased from 4.55 ± 3.02 × 10^−3^ to 2.7 ± 1.83 × 10^−3^ Ω/Sq for 18-nm Au film and from 2.66 ± 3.4 × 10^−3^ to 1.94 ± 1.28 × 10^−3^ Ω/Sq for 39-nm film for green laser. After UV laser scanning, it reduced from 5.26 ± 3.2 × 10^−3^ to 3.4 ± 2.89 × 10^−3^ Ω/Sq for 18-nm and from 2.58 ± 2.84 × 10^−3^ to 1.73 ± 2 × 10^−3^ Ω/Sq. for 39-nm thick Au film, respectively.

For 18-nm thick Au films, the average electrical conductivity is improved up to 40% for green, 35% for UV, and 33% for IR laser-treated films whereas, for 39-nm film, an average improvement of the order of 29%, 27% and 15% for UV, green and IR laser wavelength is obtained, respectively. We infer that there exists a relationship between the laser wavelength and the degree of crystallization which also depends upon film thickness. Our experimental findings reveal that 18-nm films were improved significantly more than 39-nm thick Au films. The green laser is more effective for 18-nm film, as film thickness lies within the absorption length which causes a homogenous heating of overall film, whereas UV laser triggered more crystallization in 39-nm Au films due to significant inter-band and intra-band absorption.

### 3.3. AFM Measurements

**Figure 5:**
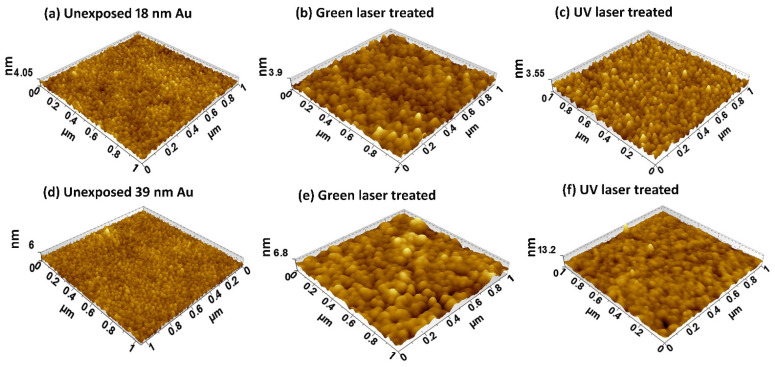
Topographic AFM images of (**a**) as deposited 18-nm thick Au film and (**b**,**c**) after laser scanning while (**d**) is as-deposited 39-nm thick film and (**e**,**f**) are after scanning with green and UV laser pulses, respectively.

AFM measurements are performed to investigate the surface morphologies of as deposited and laser-scanned Au films. Figure 5 represents topographical images of 18-nm (5a–5c) and 39-nm (5d–5f) Au films on quartz substrate before and after green and UV laser scanning. The AFM images agree well with electrical results as significant enhancement in particle size can be seen in Figure 5. The AFM images of 1 × 1 µm^2^ area are selected randomly on 9 mm × 9 mm samples. The as deposited Au films are composed of tiny particles with an average lateral particle size of 19 nm for 18-nm and 21 nm for 39-nm film, respectively. After scanning with green laser pulses (Figure 5b,e), these tiny particles agglomerate into bigger particles and the average lateral particle size increases to 35 nm for 18-nm thick film and to 47 nm for 39-nm thick Au film on quartz substrate. The higher optical penetration depth of green laser compared to UV and IR wavelengths results in a vertical growth direction along with the lateral one due to higher absorption of laser energy deeper into the film thickness. Similarly, when scanned with UV laser pulses, the particle size increases to 25 nm for 18-nm film and 26 nm for 39-nm thick Au film, respectively. In case of UV laser wavelength, the energy absorbs in surface and the particle growth in lateral direction occurs which is more pronounced in Figure 5f. The particles are closer to each other and enhance the surface smoothness. The surface roughness of as deposited 18-nm Au films on quartz substrate is 0.48 nm. After crystallization, surface roughness reduces to 0.46 nm for green laser and 0.42 nm for UV laser, respectively. The surface roughness of untreated 39 nm Au film is 0.54 nm which increased to 0.81 nm for green laser treated films and reduced to 0.78 nm for the UV laser-scanned sample. The higher value of surface roughness (0.78 nm) in case of UV laser treated 39-nm Au film is due to appearance of few particles of greater height. Note that the surface roughness of the bare quartz substrate was 0.35 nm.

### 3.4. XRD Analysis

**Figure 6:**
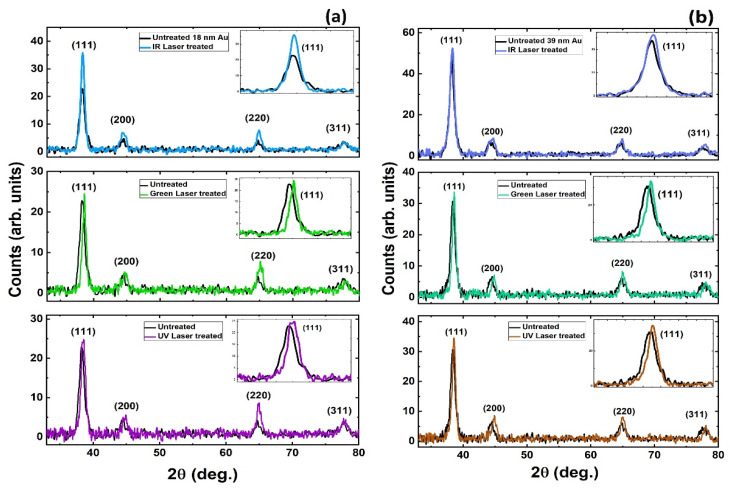
XRD spectra of thin films before and after laser scanning for (**a**) 18-nm and (**b**) 39-nm thick Au films on quartz substrate.

In XRD spectra, the full width at half maximum (FWHM) of peaks are related to grain size of the film. Smaller FWHM means larger grains and hence, better crystalline nature of the film [31]. Since our Au films were initially polycrystalline, a sharp peak and reduced FWHM indicates an improved crystal quality after laser scanning. The scattered peaks are identified as 111, 200, 220 and 311 (Figure 6). In case of 18-nm Au film, peak (111) shows a decrease in FWHM by 19% centered at 38.28° Bragg’s angle for IR, 25% decrease for green, 23% decrease for UV laser, respectively. Similarly, the FWHM is reduced by 5% for IR, 22% for green and 23.5% for UV laser treated 39-nm thick Au films. A slight red shift in the 111 peak occurred after laser crystallization due to relaxation of internal compressive stresses (strains) formed during thin film deposition [31]. The Scherrer equation is used to estimate the size (*L*) of nano-crystallites as [32];

(2) $$L\left(nm\right)=\frac{0.94\;\left(Scherrer\;constant\right)\;\lambda \;(CuKa\;1.94\;Angstrom)}{\beta cos\theta \;(radian)}$$

where β is FWHM at diffracted angle (2 θ). The estimated crystallite sizes are in agreement with the AFM measurements of particle sizes and overall particle/crystallite sizes which were improved after laser scanning (Table 2). XRD results indicate that crystallization occurred in the laser-scanned Au thin films.

### 3.5. Optical Properties

**Figure 7:**
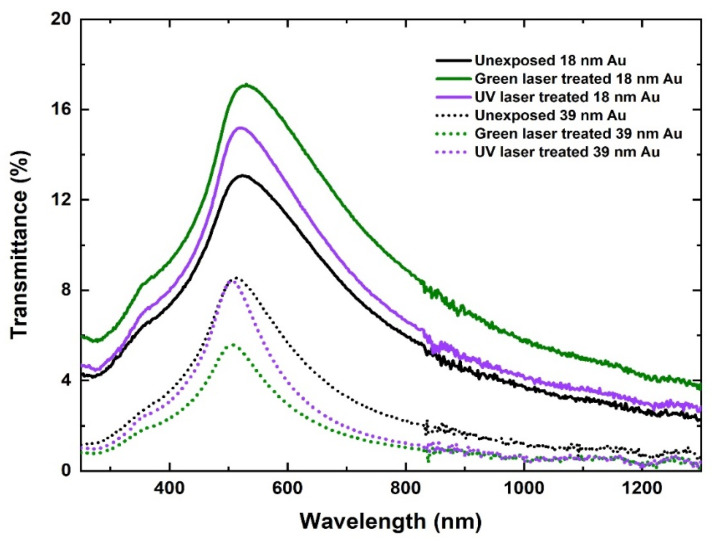
Effect of laser crystallization on optical properties of 18-nm and 39-nm thick Au films before and after green and UV laser scanning, respectively.

The effect of laser crystallization on optical properties of Au films was investigated for green and UV laser-scanned samples, as these wavelengths were capable of triggering interband absorption in Au. Figure 7 represents the transmittance spectra for 18-nm and 39-nm thick Au films after green and UV laser scanning. An increase in transmittance is observed for 18-nm film from 13.08% (as deposited) to 17.13% (green) and 15.2% (UV) for laser-scanned samples, respectively. The maximum transmittance depends on the type of the material and for Au it is observed in the visible region. Au thin films have a maximum transmittance at λ ≈ 500 nm which, is evident in Figure 7 [33]. This peak is slightly blue shifted for thick films (39 nm) due to increased carrier density of the film. Transmittance also depends on the degree of crystallization as the higher the number of grain boundaries the larger the scattering, resulting in lower optical transmittance [34]. The increase in grain size after laser scanning causes a decrease in the number of grain boundaries. Therefore, the increase in optical transmission after the laser scanning in 18-nm films is due to the improved crystallinity as confirmed by AFM and XRD characterizations. The optical absorption of Au in the visible region is due to relativistic decrease in the gap between the 5d band and the Fermi level indicated by the low interband transition threshold (1.84 eV). The transmission falls at higher wavelengths due to the interaction of laser light with high numbers of electrons which reflect the light in the IR region. Intraband and interband absorption occurs in the UV region due to strong absorption of laser photons by free carriers in Au thin films. However, in the case of 39-nm thick Au films, transmittance decreases slightly from 8.56% to 8.42% for green laser and to 5.58% for UV laser-treated films, respectively. This decrease is due to higher film thickness and increased particle densities where the improved grain sizes are responsible for this decrease in the laser annealed 39-nm thick Au films.

The experimental results obtained are promising and confirm a low temperature crystallization process in Au thin films. No melting was observed on film surfaces nor any damage, since the fluence range was kept below the damage threshold. A numerical simulation study was performed to investigate the fs laser interactions with Au thin film and the following non-equilibrium energy exchange between electrons and the lattice system. The numerical results support a non-thermal crystallization process since the fluence used was unable to cause any melting (Section 4).

## 4. Numerical Modelling

### Two Temperature Gold-Quartz Interface Model

COMSOL Multiphysics^®^ software was used to model the response and interplay of 18-nm Au thin film on a quartz substrate when treated by 1030-nm, 500-fs ultrashort laser pulses. It is important to understand energy transfer from laser excited electrons withing thin metal film and to the dielectric substrate by considering Au-quartz interface into the model. A striking feature of ultrashort laser mater interaction is that only electrons are excited by photon–electron interactions within the pulse duration and become hot by establishing an electron temperature *T_e_* where the lattice is considered as cold; the lattice temperature *T_l_* is unchanged. This results in extremely high temperature gradients between electrons and the lattice, creating two temperatures (*T_e_* and *T_l_*) within the material. This non-equilibrium heat transfer can be described by the two-temperature model (TTM) as in [17,35]

(3) $$C_{e}\left(T_{e}\right)\frac{\partial T_{e}}{\partial t}=\nabla \left(k_{e}\nabla T_{e}\right)-G\left(T_{e}-T_{l}\right)+S\left(x,\;t\right),$$

(4) $$C_{l}\left(T_{l}\right)\frac{\partial T_{l}}{\partial t}=\nabla \left(k_{l}\nabla T_{l}\right)+G\left(T_{e}-T_{l}\right)$$

where subscripts (*e*, *l*) denote the electron and lattice subsystem. *C*, *T*, and *k* represent volumetric heat capacity (Jm^−3^K^−1^), temperature (K) and thermal conductivity (Wm^−1^K^−1^), respectively. (Wm^−3^ K^−1^) is an temperature dependent electron–phonon coupling factor and accounts for the rate of energy transfer from hot electrons to the cold lattice. The laser energy was Gaussian distribution over both time and space and the volumetric laser source term *S(x,t)* can be written as [17];

(5) $$S\left(r,t\right)=\frac{(\alpha \times 0.94\times \phi_{o}\times \left(1-R\right)}{t_{p}}\;exp\left[\frac{-2x^{2}}{\omega_{o}^{2}}-4ln2{\left(\frac{t-t_{r}}{t_{p}}\right)}^{2}\right]exp\left(-\alpha z\right)$$

where α is the absorption coefficient (m^−1^), $\phi_{o}$ is applied laser fluence (Jcm^−2^), *R* is reflectivity, $\omega_{o}$ is spot radius, *t_r_* is reference time and *t_p_* is the laser pulse duration.

In metals, electrons are responsible for heat conduction, whereas phonons dominate heat conduction in semiconductors and dielectric materials [36]. As the quartz is a dielectric with no free electrons, there could be two possible mechanisms for heat transfer across the metal–nonmetal interface: (i) through coupling between metal electrons and substrate phonons with an interfacial resistance *R_es_* and (ii) through coupling between metal electrons and metal phonons and then heat transfer by metal phonons to phonons in the substrate with interfacial resistance *R_ls_* [36]. A third equation is introduced into the model to describe thermal conduction from the Au film to a quartz substrate as [35]

(6) $$C_{s}\;\frac{\partial T_{s}}{\partial t}=\nabla \left(k_{s}\nabla T_{s}\right),$$

where *T_s_* is lattice temperature of substrate, *C_s_* and *k_s_* are heat capacity and thermal conductivity of substrate. The interface boundary conditions can be written as [35]

(7) $$-k_{e}{\frac{\partial T_{e}}{\partial x}|}_{x=L}={\frac{T_{e}-T_{s}}{R_{es}}|}_{x=L}$$

(8) $$-k_{l}{\frac{\partial T_{l}}{\partial x}|}_{x=L}={\frac{T_{l}-T_{s}}{R_{ls}}|}_{x=L}$$

(9) $$-k_{s}{\frac{\partial T_{s}}{\partial x}|}_{x=L}={\frac{T_{e}-T_{s}}{R_{es}}|}_{x=L}+{\frac{T_{l}-T_{s}}{R_{ls}}|}_{x=L}$$

*R_es_* is thermal resistance between Au electrons and substrate phonons; *R_ls_* is the resistance between Au phonons and phonons in the substrate and L is the film thickness. The temperature dependent electron heat conductivity can be written as [37]

(10) ke=χ(φe2+0.16)54 (φe2+0.44)φe(φe2+0.092)12 (φe2+ηφl) ,

(11) $$\varphi_{e}=\frac{T_{e}}{T_{F}},\;\mathrm{and}\;\varphi_{l}=\frac{T_{l}}{T_{F}},$$

where *T_F_* is the Fermi temperature of Au and χ and η are material dependent constants. Temperature dependent *C_e_* and *G(T)* are taken from [38]. Thermophysical parameters used in the simulation are listed in Table 3 and the initial temperature in the simulation was kept at 300 K.

When an ultrashort laser is irradiated on a metal surface, the energy is absorbed by the free carriers in metals through photon–electron interactions and the electronic subsystem changes from ground state to the excited state. After few fs electrons re-establish the Fermi–Dirac distribution and the characteristic time required by the electrons to restore their states is called the electron relaxation time [17]. Initially the excited electrons are localized within the optical absorption depth (12.5 nm for IR) and diffuses into the deeper parts as a result of large temperature gradients in the system. As the electron heat capacity is typically orders of magnitude lower than lattice, the electrons temperature (*T_e_*) reaches up to thousands of kelvins where the lattice temperature (*T_l_*) is almost unperturbed (*T_e_ > T_l_*). After picoseconds (ps), the thermal energy is then transferred to the lattice by means of electron–phonon interactions until a thermal equilibrium is achieved.

**Figure 8:**
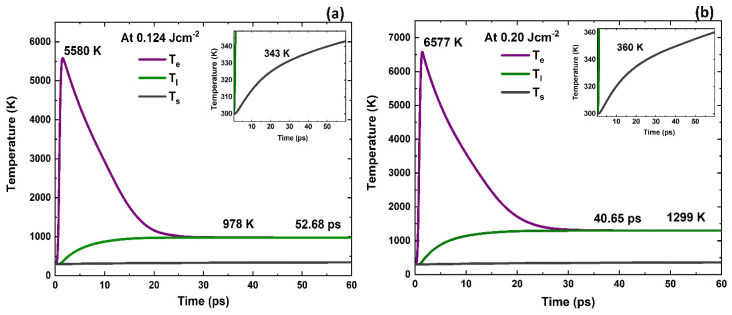
Surface temperature profiles as a function of time for 18-nm thick Au film on quartz, irradiated by 500 fs, 1030 nm pulse at (**a**) 0.124 Jcm^−2^ and (**b**) 0.20 Jcm^−2^, respectively.

The simulation results describing the evolution of electron and lattice temperatures for 18-nm Au film at quartz substrate are presented in Figure 8 for the IR femtosecond laser wavelength. Figure 8a shows the temporal distribution of electron temperature, lattice temperature at the Au film surface and center of the laser beam (*x =* 0, *z* = 0) and substrate temperature at the Au–quartz interface (*x* = 0, *z* = 18-nm). Laser fluence of 0.124 Jcm^−2^ ($\phi_{th}$ for 18-nm film) was used in simulating electron and lattice temperature evolution as a result of laser interaction with Au thin film. The model predicts that *T_e_* rises sharply due to small heat capacities of electrons and reaches a maximum of 5580 K at 1.49, ps whereas *T_l_* rises at a slower rate. Electrons start to thermalize by transferring their energies to other electrons and with the lattice through electron–phonon interactions. As a result, *T_e_* starts decreasing and *T_l_* starts rising (Figure 8a). The thermalization time at which electrons and lattice establish an equilibrium is 52.68 ps. *T_l_* rises to a maximum of 978 K, less than the melting temperature of Au (1337.33 K) [17]. The substrate temperature (*T_s_*) also increases but at a slower rate and increases from 300 K to 343 K (Figure 8a inset).

The simulation was performed at a fluence (0.20 Jcm^−2^) relatively higher than $\phi_{th}$ in order to observe melting (Figure 8b). It is observed that *T_e_* reaches 6577 K at 1.41 ps (Figure 8b). *T_l_* increases up to 1299 K, still lower than melting temperature of Au but, if a higher value of fluence is applied, it will result in the melting of the lattice. In the case of *T_s_,* temperature varies from 300 K to 360 K at 0.20 Jcm^−2^ applied fluence and electrons completely thermalize their energies with the lattice at 40.65 ps. At both fluences of 0.124 Jcm^−2^ and 0.20 Jcm^−2^, *T_l_* remained below the melting point of Au suggesting that such low fluences were unable to cause any melting in the system. From these computation results, we infer that our crystallization process is a non-melt process. Upon laser irradiation, electrons are excited first and such excitation can modify the electron density distribution in the solid, giving rise to modified forces between atoms and can directly affect the order of the lattice. The modified interatomic forces in turn cause coherent atomic motion and structural transitions on a very short time scale (sub-picosecond) [39]. Such transitions occur without electron to phonon energy transfer (picoseconds) and are called non-thermal phase transitions. We suggest that crystallization occurred as a result of non-thermal solid-state diffusion of atoms in interstitial sites at a local scale of nanometers.

**Table 3:** Thermophysical properties of gold and quartz used in the model.

Property	Gold	Quartz (SiO_2_)
Melting temperature T_melt_ (K)	1337.33 [17]	1943 [29]
Mass density *ρ* (Kgm^−3^)	19320 [40]	2620 @ 293 K [29]
Fermi temperature T_F_ (K)	6.42 × 10^4^ [41]	-----
Lattice heat capacity C_l_ (Jm^−3^K^−1^)	2.5 × 10^6^ [42]	1.93 × 10^6^ [43]
Thermal heat conductivity *k_l_* (Wm^−1^K^−1^)	317 @ 300 K [44]	13.93 @ 300 K [43]
Absorption coefficient α (m^−1^)	8.178 × 10^7^ @ 1030 nm [24]	0.725 @ 1030 nm [45]
Reflectance *R*	0.97 @ 1030 nm [24]	0.8821 @ 1030 nm [46]
Constants χ (Wm^−1^K^−1^) and η for equation 10	353, 0.16 [37]	
*R_ls_* (m^2^K W^−1^)		2.5 × 10^−8^ [47]

## 5. Conclusions

We investigated a low temperature Au thin film crystallization by scanning with highly overlapped fs laser pulses of three different harmonics. The effect of laser wavelengths is explored for a laser induced damage threshold $\left(\phi_{th}\right)$ and laser induced crystallinity on two Au films thicknesses deposited on a 500-µm quartz substrate. The damage threshold is significantly influenced by the laser wavelength and film thickness. Up to 40% improvements in crystallinity are achieved for 18-nm films with green laser and 29% in 39-nm Au films with UV laser, respectively. Surface characterization and a two-temperature Au–quartz interface model suggest that the proposed low fluence based crystallization process is melt-free and can be applied in crystallization of Au film on a heat sensitive substrate.

The proposed low temperature process in the current study for metal (Au) films crystallization using ultra-short laser pulses is also proven to be promising, as has been demonstrated for crystallizing indium doped tin oxide (ITO) thin films [34]. This method is unique in providing selective and localized crystallization with high precision without damaging the substrate and any nearby heat-sensitive surfaces or components. It could be highly relevant in improving the electrical properties of thin film-based electrochemical and optical-based sensors for signal enhancement applications. Our process is significant as it enables the reduction of deposition and annealing temperatures to produce conductive gold thin films for their better application in medical, commercial, and industrial disciplines where surface functionality is a key factor.

## Figures and Tables

**Table 1:** Experimentally calculated parameters for 18-nm and 39-nm Au films on quartz substrates for a 500-fs laser operating at 100 kHz repetition rate.

Laser Wavelength	Absorption Length *l_opt_* [24]	Beam Diameter 2 ω_o_	SPA	Pulse Overlap	$\mathrm{Damage}\;\mathrm{Threshold}\;\phi_{th}$	
					18 nm	39 nm
nm	nm	µm		%	Jcm^−2^	Jcm^−2^
IR (1030)	12.50	43.9	24.39	96	0.124 ± 8.11 × 10^−4^	0.323 ± 8.11 × 10^−4^
Green (515)	20.27	35.7	19.86	95	0.025 ± 7.07 × 10^−4^	0.056 ± 8.37 × 10^−4^
UV (343)	14.59	29.0	16.12	93.8	0.026 ± 5.30 × 10^−4^	0.042 ± 3.80 × 10^−4^

**Table 2:** Estimated crystallite size from XRD analysis and corresponding improvements in crystallinity for IR, green and UV laser, respectively.

Gold Films	Untreated	Laser Treated					
Thickness		IR (1030 nm)		Green (515 nm)		UV (343 nm)	
(nm)	L (nm)	L (nm)	Crystallinity	L (nm)	Crystallinity	L (nm)	Crystallinity
18	9.10	11.05	33%	12.09	40%	11.80	35%
39	9.24	9.82	15%	12.8	27%	12.99	29%

## Data Availability

The data presented in this study are available on request from the corresponding author.

## References

[1] Amendola V., Pilot R., Frasconi M., Maragò O.M., Iatì M.A. (2017). Surface plasmon resonance in gold nanoparticles: A review. J. Phys. Condens. Matter..

[2] Elahi N., Kamali M., Baghersad M.H. (2018). Recent biomedical applications of gold nanoparticles: A review. Talanta.

[3] Toudeshkchoui M.G., Rabiee N., Rabiee M., Bagherzadeh M., Tahriri M., Tayebi L., Hamblin M.R. (2019). Microfluidic devices with gold thin film channels for chemical and biomedical applications: A review. Biomed. Microdevices.

[4] Daggumati P., Matharu Z., Seker E. (2015). Effect of nanoporous gold thin film morphology on electrochemical DNA sensing. Anal. Chem..

[5] Qi H., Niu L., Zhang J., Chen J., Wang S., Yang J., Guo S., Lawson T., Shi B., Song C. (2018). Large-area gold nanohole arrays fabricated by one-step method for surface plasmon resonance biochemical sensing. Sci. China Life Sci..

[6] Lansåker P.C., Petersson P., Niklasson G.A., Granqvist C.-G. (2013). Thin sputter deposited gold films on In_2_O_3_: Sn, SnO_2_: In, TiO_2_ and glass: Optical, electrical and structural effects. Sol. Energy Mater. Sol. Cells.

[7] Chang J., Young T., Yang Y., Ueng H., Chang T. (2004). Silicide formation of Au thin films on (1 0 0) Si during annealing. Mater. Chem. Phys..

[8] Sun X., Li H. (2013). Gold nanoisland arrays by repeated deposition and post-deposition annealing for surface-enhanced Raman spectroscopy. Nanotechnology.

[9] Dishner M.H., Ivey M.M., Gorer S., Hemminger J.C., Feher F.J. (1998). Preparation of gold thin films by epitaxial growth on mica and the effect of flame annealing. J. Vac. Sci. Technol. A Vac. Surf. Film..

[10] Do M.T., Tong Q.C., Lidiak A., Luong M.H., Ledoux-Rak I., Lai N.D. (2016). Nano-patterning of gold thin film by thermal annealing combined with laser interference techniques. Appl. Phys. A.

[11] Duley W.W. (2012). Laser Processing and Analysis of Materials.

[12] Arnob M.M.P., Zhao F., Zeng J., Santos G.M., Li M., Shih W.-C. (2014). Laser rapid thermal annealing enables tunable plasmonics in nanoporous gold nanoparticles. Nanoscale.

[13] Wu T.-H., Kalim S., Callahan C., Teitell M.A., Chiou P.-Y. (2010). Image patterned molecular delivery into live cells using gold particle coated substrates. Opt. Express.

[14] Kumar P., Krishna M.G. (2010). A comparative study of laser-and electric-field-induced effects on the crystallinity, surface morphology and plasmon resonance of indium and gold thin films. Phys. Status Solidi A.

[15] Bäuerle D. (2011). Laser Processing and Chemistry.

[16] Gamaly E.G. (2011). Femtosecond Laser-Matter Interaction: Theory, Experiments and Applications.

[17] Jiang L., Tsai H.-L. (2005). Improved two-temperature model and its application in ultrashort laser heating of metal films. J. Heat Transfer..

[18] Zhan X.-P., Hou M.-Y., Ma F.-S., Su Y., Chen J.-Z., Xu H.-L. (2019). Room temperature crystallization of amorphous silicon film by ultrashort femtosecond laser pulses. Opt. Laser Technol..

[19] Hoppius J.S., Bialuschewski D., Mathur S., Ostendorf A., Gurevich E.L. (2018). Femtosecond laser crystallization of amorphous titanium oxide thin films. Appl. Phys. Lett..

[20] Chen S.-C., She N.-Z., Juang J.-Y., Chen Y.-Z., Kuo H.-C., Chueh Y.-L., Wu K.-H. (2017). Femtosecond Laser Crystallization for Boosting the Conversion Efficiency of Flexible Ink-Printing Cu (In, Ga) Se2 Thin Film Solar Cells. CLEO: Applications and Technology, San Jose, CA, USA, 14–19 May 2017.

[21] Zhang G., Gu D., Gan F., Jiang X., Chen Q. (2005). Femtosecond laser-induced crystallization in amorphous Ge2Sb2Te5 films. Thin Solid Film.

[22] Katayama S., Tsutsumi N., Nakamura T., Horiike M., Hirao K. (2002). Femtosecond laser induced crystallization and permanent relief grating structures in amorphous inorganic (In 2 O 3+ 1 wt% TiO 2) films. Appl. Phys. Lett..

[23] Liu J. (1982). Simple technique for measurements of pulsed Gaussian-beam spot sizes. Opt. Lett..

[24] Johnson P.B., Christy R.-W. (1972). Optical constants of the noble metals. Phys. Rev. B.

[25] Krüger J., Dufft D., Koter R., Hertwig A. (2007). Femtosecond laser-induced damage of gold films. Appl. Surf. Sci..

[26] Wellershoff S.-S., Hohlfeld J., Güdde J., Matthias E. (1999). The role of electron–phonon coupling in femtosecond laser damage of metals. Appl. Phys. A.

[27] Haustrup N., O’Connor G. (2012). Impact of wavelength dependent thermo-elastic laser ablation mechanism on the generation of nanoparticles from thin gold films. Appl. Phys. Lett..

[28] Catherine L., Olivier P. (2017). Gold Nanoparticles for Physics, Chemistry and Biology.

[29] Haustrup N. (2014). Wavelength Dependence of Femtosecond Laser Ablation of Thin Gold Films. Ph.D. Thesis.

[30] Zhang X., Huang C., Wang M., Huang P., He X., Wei Z. (2018). Transient localized surface plasmon induced by femtosecond interband excitation in gold nanoparticles. Sci. Rep..

[31] Shim E.S., Kang H.S., Pang S.S., Kang J.S., Yun I., Lee S.Y. (2003). Annealing effect on the structural and optical properties of ZnO thin film on InP. Mater. Sci. Eng. B.

[32] Klug H.P., Alexander L.E. (1974). X-ray diffraction procedures: For polycrystalline and amorphous materials. X-ray Diffraction Procedures: For Polycrystalline and Amorphous Materials.

[33] Axelevitch A., Gorenstein B., Golan G. (2012). Investigation of optical transmission in thin metal films. Phys. Procedia.

[34] Farid N., Sharif A., Vijayaraghavan R., Wang M., Chan H., Brunton A., McNally P., Choy K., O’Connor G. (2021). Improvement of electrical properties of ITO thin films by melt-free ultra-short laser crystallization. J. Phys. D Appl. Phys..

[35] Yao Q., Guo L., Iyer V., Xu X. (2019). Ultrafast Electron–Phonon Coupling at Metal-Dielectric Interface. Heat Transf. Eng..

[36] Majumdar A., Reddy P. (2004). Role of electron–phonon coupling in thermal conductance of metal–nonmetal interfaces. Appl. Phys. Lett..

[37] Chen J., Latham W., Beraun J. (2005). The role of electron–phonon coupling in ultrafast laser heating. J. Laser Appl..

[38] Lin Z., Zhigilei L.V., Celli V. (2008). Electron-phonon coupling and electron heat capacity of metals under conditions of strong electron-phonon nonequilibrium. Phys. Rev. B.

[39] Letfullin R.R., George T.F., Duree G.C., Bollinger B.M. (2008). Ultrashort laser pulse heating of nanoparticles: Comparison of theoretical approaches. Adv. Opt. Technol..

[40] Zhang Y., Chen J. (2007). Melting and resolidification of gold film irradiated by nano-to femtosecond lasers. Appl. Phys. A.

[41] Majchrzak E., Dziatkiewicz J. (2012). Application of the two-temperature model for a numerical study of multiple laser pulses interactions with thin metal films. Sci. Res. Inst. Math. Comput. Sci..

[42] Kanamori H., Fujii N., Mizutani H. (1968). Thermal diffusivity measurement of rock-forming minerals from 300° to 1100° K. J. Geophys. Res..

[43] Ho C.Y., Powell R.W., Liley P.E. (1972). Thermal Conductivity of the Elements. J. Phys. Chem. Ref. Data.

[44] Khashan M., Nassif A. (2001). Dispersion of the optical constants of quartz and polymethyl methacrylate glasses in a wide spectral range: 0.2–3 μm. Opt. Commun..

[45] Coblentz W.W. (1915). Absorption, Reflection, and Dispersion Constants of Quartz.

[46] Losego M.D., Grady M.E., Sottos N.R., Cahill D.G., Braun P.V. (2012). Effects of chemical bonding on heat transport across interfaces. Nat. Mater..

[47] Giret Y., Daraszewicz S.L., Duffy D.M., Shluger A.L., Tanimura K. (2014). Nonthermal solid-to-solid phase transitions in tungsten. Phys. Rev. B.

